# Gender profile of principal investigators in a large academic clinical trials group

**DOI:** 10.3389/fsurg.2022.962120

**Published:** 2022-07-18

**Authors:** Vi Thi Thao Luong, Cindy Ho, Veronica Aedo-Lopez, Eva Segelov

**Affiliations:** ^1^ Oncology Department, Monash Health, Melbourne VIC Australia; ^2^ School of Clinical Sciences, Monash University, Melbourne, VIC, Australia

**Keywords:** gender equity, female surgeon, principal investigator, clinical trials, surgical oncologist, leadership

## Abstract

**Introduction:**

Gender equity in medicine has become a significant topic of discussion due to consistently low female representation in academia and leadership roles. Gender imbalance directly affects patient care. This study examined the gender and craft group of the Principal Investigators (PI) of clinical trials run by the Australasian Gastro-Intestinal Trials Group (AGITG)

**Methods:**

Publicly available data was obtained from the AGITG website. Trials were divided into upper, lower gastrointestinal cancer, miscellaneous (neuroendocrine and gastrointestinal stromal tumours). Where multiple PIs were listed, all were counted. Craft group was assigned as surgical, medical, radiation oncology or other.

**Results:**

There were 69 trials with 89 PI, where 52 trials were represented exclusively by male PIs. Of all PIs, 18 were women (20.2%); all were medical oncologists. Prior to 2005, all PIs were male. The craft group distribution of PIs was: 79% medical oncologists, 12% surgical oncologists, 8% radiation oncologist, 1% nuclear medicine physicians. Regarding trials with multiple PI's, there were 19 in total. Of these, 11 had only male PIs, which included 5 surgeons. Females were more likely to be a co-PI (42%) as opposed to sole PI (18%). There was no gender policy publicly available on the AGITG website.

**Conclusions:**

There is a low percentage of female PIs in academic oncology trials in the portfolio of this large international trials group. No trial was led by a female surgical or radiation oncologist. There is a need to understand the reasons driving the disparity so that specific strategies can be put in place.

## Introduction

Gender inequity in medicine remains a global issue, despite many years of policies and initiatives to promote participation and progression to leadership roles. Women constitute the majority of the medical workforce, yet are still grossly under-represented in many specialties and at senior levels (1). Gender bias is proven to impact adversely on patient care (2). Diversity, including in gender, brings broadening of values, opinions and collective contributions and most importantly, reflects the broad community, who are after all the main stakeholder in healthcare provision.

It is widely recognised that females are under-represented in almost every country across the spectrum of surgical subspecialties (1, 3–5). A recent systematic review demonstrated a self-perpetuating cycle, where lack of progression in career development of female surgeons is shown to perpetuate the imbalance (6). Additionally, gender equity statements and policies among professional surgical societies were recently catalogued as deficient (7). To date there is little data available with respect to the surgical oncology craft group. Other oncology disciplines such as medical oncology, haematology and radiation oncology have shown increased female representation over time, although imbalances remain in various proportions (1, 4, 8, 9).

Despite the rise in female presence by numbers, this has not translated to equity at the level of senior positions, such as decision-making or developmental roles, both within clinical care and academia (10–17). The imbalance is even stronger in certain geographical regions, such as Asia-Pacific, and has been exacerbated by the COVID-19 pandemic (18–20). Over the past few years, growing numbers of reports of gender distribution in specific leadership positions and roles have been published, including analysis of first and senior authors in publications; journal editorial boards; presenters at major meetings and even the differences by gender of the use of titles by chairpersons when introducing speakers at international meetings (21–25).

One problem is that researching the topic of gender equity is often met with hostility and researchers can be ostracised (26, 27). By contrast, it is only when this data becomes welcomed that there is likely to be a culture in which positive change can be achieved.

In Australia, data regarding gender equity in the various disciplines of oncology is lacking. As an exemplar, we studied leadership in the field of clinical trials by examining the gender of trial Principal Investigators (PI). The PI plays a pivotal role in establishing, running and reporting a trial, leading the Trial Management Committee and liaising with trial investigators. Most oncology trials have a sole PI, although increasingly the role is shared amongst two or even three individuals. This research presents the distribution and trends in PI gender in the Australasian Gastro-Intestinal Trials Group (AGITG), a large academic, international, not-for-profit, trials consortium.

## Methods

Publicly accessible areas of the AGITG website (https://gicancer.org.au/about-the-agitg/) were viewed on several occasions between July and August 2021. All listed trials were sub-grouped into categories: completed, open, in-development and in follow-up. Trials were further sorted into tumour site as: Upper Gastrointestinal (GI), Lower GI and other (gastrointestinal stromal tumour and neuroendocrine tumours). The year of trial registration and the gender of the PI(s) were recorded. Gender was confirmed from personal knowledge and/or Google® searches for public profile. PIs were counted for every trial they led; where trials had multiple PIs, each was recorded. Data was verified by a second investigator.

## Results

Across 69 clinical trials conducted by the AGITG between 1994 and 2022, there were a total of 89 PIs, with 18 trials led by females (20.2%) comprising nine unique females. All female PIs were medical oncologists; they constituted 25.7% of the total of 70 PIs from this craft group. There were no females amongst the 11 surgical oncology, 7 radiation oncology and 1 nuclear medicine PIs. Women were under-represented as PI when trials were considered according to study status and tumour site (Table 1). Trials in the “completed” and “in follow up” categories had 8% of PIs being female (registration year between 1994 and 2018, *n* = 49), whereas the newer trials in the “open” and “in preparation” categories (registration year 2016–2022, *n* = 20) had 10% female PIs. Female PIs were less likely to be the sole PI (*n* = 9, 18% of 50 sole PI studies) than to play a role as a co-PIs (*n* = 8, 42% of the 19 co-led trials) (Table 2).

**Table 1:** Distribution of principal investigators for AGITG trials.

		Principal investigators (*n *= 89)		
	All studies (*n* = 69)	Total investigators	Males (%)	Females (%)
		89	71 (79.8)	18 (20.2)
Craft groups	Medical oncology	70	52 (74.3)	18 (25.7)
	Radiation oncology	7	7 (100.0)	0 (0.0)
	Surgical oncology	11	11 (100.0)	0 (0.0)
	Nuclear medicine	1	1 (100.0)	0 (0.0)
Study status	Completed (*n* = 43)	53	47 (88.7)	6 (11.3)
	In follow-up (*n* = 6)	8	6 (75.0)	2 (25.0)
	Open (*n *= 14)	21	13 (61.9)	8 (38.1)
	In preparation (*n *= 6)	7	5 (71.4)	2 (28.6)
Types of cancers	Upper GI (*n* = 30)	42	33 (78.6)	9 (21.4)
	Lower GI (*n* = 32)	39	31 (79.5)	8 (20.5)
	Others (NET and GIST) (*n* = 7)	8	7 (87.5)	1 (12.5)

*Abbreviation: GI, gastrointestinal, NET, neuroendocrine tumour; GIST, gastro-intestinal stromal tumour.*

**Table 2:** Principal investigator gender according to composition of PIs within AGITG.

	Sole PI (%)	2 PIs (%)	3 PIs (%)
	Studies (*n *= 50)	Studies (*n* = 18)	Studies (*n *= 1)
Male PI only	41 (82.0)	11 (61.1)	0 (0.0)
Female PI only	9 (18.0)	0 (0.0)	0 (0.0)
Mixed PIs	Not applicable	1M:1F = 7 (38.9)	1M:2F = 1 (100.0)

*Definition: Sole PI: a study that had only one PI (male or female).*

*Abbreviation: M, Male; F, Female.*

**Figure 1:**
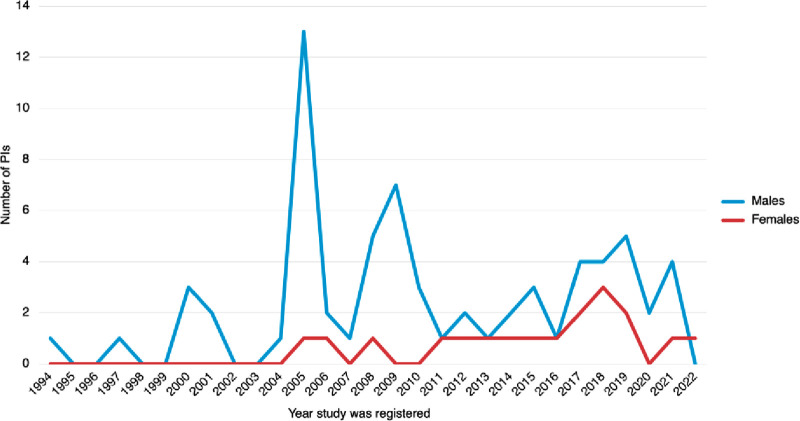
Trend over time of principal investigators within AGITG. Each trial is represented once, in the year it was registered.

The trend of male and female investigators over a nearly 30-year timeframe is shown in Figure 1. Half of all studies (*n* = 33) were conducted from 1994 to 2010, with only 7% of female investigators in this period; there were none prior to 2005. Between 2011 and 2022, female investigators constituted 34%. Our study estimated a rise of 0.6 PI per 10-year from 2015 onward.

## Discussion

Despite often being poorly received, there is a growing movement to present data about gender disparity to try to stimulate change based on evidence. We have shown a lack of female leadership in GI oncology clinical trials within a large academic organization, with the interesting (and actionable) analysis that it is female PIs particularly from surgical and radiation oncology that should be encouraged. Not only were there many less females, those who had PI roles only comprised only a few different individuals across multiple studies. This suggests that women have difficulty in “penetrating the scene” and that only a few females were offered opportunities, potentially relating to the fact that they have had to recurrently “prove their worth”.

Furthermore, significantly fewer studies within AGITG were represented by surgical oncology compared to medical oncology. This can be explained and is consistent with data presented by Wong et al where only 7.6% of surgical oncology trials from 2008 to 2020 involved surgical interventions (28). Is this because academic productivity (number of research publications and grants) as a promotion benchmark is not as highly valued and less commonly sought by employers within surgical oncology, at least in the Australian context and likely in many other countries (29)? It has been suggested that heavy workload and long hours of the surgical career lead to reduce commitment to research (30). In fact, protected research time was one of the challenges to achieving academic success (followed by academic mentorship) (31). This is a call out not only for a revised surgical training program but also increased research support and mentorship for young surgeons.

At the time of our study, publicly available data (June 2021) from the Australian Health Practitioner Regulation Agency (AHPRA) reported that 13.4% of the total of 6,445 registered surgeons were female (4). The number involved in surgical oncology is likely to be much smaller; no data is available. Of the 442 total registered radiation oncologists, 44.1% were female. It is sobering that public data prior to 2019 on surgical and radiation oncology registration by gender is unavailable; even more surprising is that there is no data even now on the proportion of female medical oncologists. Over the 3 year period from 2019 to 2021, there was only a 1% increase in total numbers of both female surgeons and radiation oncologists registered in Australia, which is roughly consistent with data from the USA (32). This indicates that the ingredients needed for significant increases have not been addressed and it is not simply a matter of time before gender is equalised.

The need to ensure balanced recruitment of patients for trials in all disciplines of medicine (and for that matter laboratory animals for pre-clinical research) has given rise to the Sex and Gender Equity in Research (SAGER) guidelines, amongst others (33–35). The phenomenon of female under-representation as clinical trial PIs and academic leaders similarly cuts across all areas of medicine, surgery and medical research (36–38).

There is scant data available regarding female oncology trial PIs. One study captured data on trials published between 2003 and 2018 with an estimated rise of 1.2% in female authors annually. Their data showed no female corresponding authors for surgical trials and a lower rate of female authors within GI and genitourinary cancer trials (39). There was a higher percentage (still less than 50%) for breast and gynecological cancers (39, 40). Jou et al reported a 3% rise annually in female PIs within phase 3 gynecological clinical trials from 2010 to 2020 internationally and as in our study, women were more likely to be leading trials with multiple co-PIs than with one PI (41).

Data on the constitution of AGITG members by gender is not publicly available and we would encourage transparency in this, so that the “talent pool” from which PIs are nurtured can be understood. Additionally, at the time of the study, AGITG did not have a publicly available policy on gender equity, nor a description of the process by which trial PIs are selected. As a non-governmental organization with a significant fundraising mission and profile, we respectfully suggest that these policies be made available. However, having a policy is just the starting point, although it lays a benchmarking process that can be actively monitored. Translating the ideas and goals of a gender equity policy into significant and sustained change is where the problem lies. In Australia, one of the largest philanthropic funders of cancer research announced this year that it would not support future projects at a major University, until such time as their gender policies were reflected in actual equitable outcomes (42).

A large survey of both male and female members of the European Society of Medical Oncology published in 2018 revealed that it is not simply a matter of proportion, but rather opportunities that encourages female leadership, such as leadership training and mentoring, facilitating work life balance and provision of a flexible working environment (43). Many systematic reviews reported that strategies such as mentoring programs, education and professional development create positive outcomes in improving women's skills, however there is a dearth of appropriate mentors (44–46). Furthermore, it can only be fundamental institutional, cultural reform and perhaps even quotas, rather than individual training, that will bring about gender equality (16, 47–49).

A limitation of our study is that we did not personally contact PIs to confirm their gender. Comparative statistical analysis was not performed because there is no equivalent Australian population of physician or trial investigator according to oncology graft groups. In addition, the number of trials per year within AGITG was small and hence the trend over time of female PI was not strengthened with statistical significance. Nevertheless, the data presented is useful to inform concrete planning for positive change. We hope that the results will be openly and positively received, rather than the defensive response that similar studies internationally often encounter (26, 27). We are currently undertaking a similar review of multiple other Australian cancer trials groups to examine the gender issue in a broader sample (50).

This study shows a lack of female leadership within a large academic clinical trials group, although improvement within one specialty (medical oncology) has been seen over time. For AGITG and most likely, many other oncology trials groups across the globe, the challenges are to rectify the lack of females from surgical and radiation oncology specialties; to expand the number of different women leading trials, to address the imbalance of females being sole lead rather than co-leads; to publish gender equity policies and then enact them, and to make transparent their guidelines for selecting trial PIs.

## Conclusion

Females are under-represented as clinical trial PIs in this large Australian academic clinical trials group. There should be a focus understanding why this discrepancy still exists in 2022 and on concrete steps to ensure a balance of PIs, particularly female surgical and radiation oncologists, as well as for a broader number of individual women.

## Data Availability

The original contributions presented in the study are included in the article/Suplementary Material, further inquiries can be directed to the corresponding author/s.

## References

[1] Colleges TAoAM. Physician Specialty Data Report 2019. Available from: https://www.aamc.org/data-reports/workforce/report/physician-specialty-data-report

[2] Silver JK Bean AC Slocum C Poorman JA Tenforde A Blauwet CA Physician workforce disparities and patient care: a narrative review. Health Equity. (2019) 3(1):360–77. 10.1089/heq.2019.0040 31312783 PMC6626972

[3] Newman TH Parry MG Zakeri R Pegna V Nagle A Bhatti F Gender diversity in UK surgical specialties: a national observational study. BMJ Open. (2022) 12(2):e055516. 10.1136/bmjopen-2021-055516 PMC8968535 35314455

[4] Agency AHPR. The public national register of practitioners. Available from: https://www.medicalboard.gov.au/news/statistics.aspx

[5] Chowdhary M Chowdhary A Royce TJ Patel KR Chhabra AM Jain S Women's representation in leadership positions in academic medical oncology, radiation oncology, and surgical oncology programs. JAMA Netw Open. (2020) 3(3):e200708. 10.1001/jamanetworkopen.2020.0708 32159809 PMC7066474

[6] Lim WH Wong C Jain SR Ng CH Tai CH Devi MK The unspoken reality of gender bias in surgery: a qualitative systematic review. PLoS One. (2021) 16(2):e0246420. 10.1371/journal.pone.0246420 33529257 PMC7853521

[7] Heisler CA Miller P Stephens EH Ton J Temkin SM. Leading from behind: paucity of gender equity statements and policies among professional surgical societies. Am J Surg. (2020) 220(5):1132–5. 10.1016/j.amjsurg.2020.06.041 32709410

[8] Choinski K Lipsitz E Indes J Phair J Gao Q Denesopolis J Trends in sex and racial/ethnic diversity in applicants to surgery residency and fellowship programs. JAMA Surg. (2020) 155(8):778–81. 10.1001/jamasurg.2020.1018 32459323 PMC7254440

[9] Abelson JS Chartrand G Moo TA Moore M Yeo H. The climb to break the glass ceiling in surgery: trends in women progressing from medical school to surgical training and academic leadership from 1994 to 2015. Am J Surg. (2016) 212(4):566–72 e1. 10.1016/j.amjsurg.2016.06.012 27649976

[10] Yalamanchali A Zhang ES Jagsi R. Trends in female authorship in major journals of 3 oncology disciplines, 2002–2018. JAMA Netw Open. (2021) 4(4):e212252. 10.1001/jamanetworkopen.2021.2252 33822071 PMC8025110

[11] Lee SF Redondo Sanchez D Sanchez MJ Gelaye B Chiang CL Wong IOL Trends in gender of authors of original research in oncology among major medical journals: a retrospective bibliometric study. BMJ Open. (2021) 11(10):e046618. 10.1136/bmjopen-2020-046618 PMC8524267 34663651

[12] Mousa M Boyle JA Teede HJ. Women physicians and promotion in academic medicine. N Engl J Med. (2021) 384(7):679–80. 10.1056/NEJMc2035793 33596371

[13] Hofstadter-Thalmann E Dafni U Allen T Arnold D Banerjee S Curigliano GMD Report on the status of women occupying leadership roles in oncology. ESMO Open. (2018) 3(6):e000423. 10.1136/esmoopen-2018-000423 30273418 PMC6157529

[14] Berghoff AS Sessa C Yang JC Tsourti Z Tsang J Tabernero J Female leadership in oncology-has progress stalled? Data from the ESMO W4O authorship and monitoring studies. ESMO Open. (2021) 6(6):100281. 10.1016/j.esmoop.2021.100281 34924143 PMC8710465

[15] Temkin SM Rubinsak L Benoit MF Hong L Chandavarkar U Heisler CA Take me to your leader: reporting structures and equity in academic gynecologic oncology. Gynecol Oncol. (2020) 157(3):759–64. 10.1016/j.ygyno.2020.03.031 32276792

[16] Ehrlich H Nguyen J Sutherland M Ali A Gill S McKenney M Gender distribution among surgical journals’ editorial boards: empowering women surgeon scientists. Surgery. (2021) 169(6):1346–51. 10.1016/j.surg.2020.12.026 33494948

[17] Jorge A Bolster M Fu X Blumenthal DM Gross N Blumenthal KG The association between physician gender and career advancement among academic rheumatologists in the United States. Arthritis Rheumatol. (2021) 73(1):168–72. 10.1002/art.41492 33460296 PMC7815955

[18] Woitowich NC Jain S Arora VM Joffe H. COVID-19 threatens progress toward gender equity within academic medicine. Acad Med. (2021) 96(6):813–6. 10.1097/ACM.0000000000003782 33003040 PMC7543905

[19] Garrido P Adjei AA Bajpai J Banerjee S Berghoff AS Choo SP Has COVID-19 had a greater impact on female than male oncologists? Results of the ESMO women for oncology (W4O) survey. ESMO Open. (2021) 6(3):100131. 10.1016/j.esmoop.2021.100131 34144778 PMC8233649

[20] European Commission, Directorate-General for Research and Innovation, Coronavirus pandemic: impact on gender equality, Publications Office (2021). https://data.europa.eu/doi/10.2777/83028.

[21] Zaza N Ofshteyn A Martinez-Quinones P Sakran J Stein SL. Gender equity at surgical conferences: quantity and quality. J Surg Res. (2021) 258:100–4. 10.1016/j.jss.2020.08.036 33002662

[22] Chatterjee P Werner RM. Gender disparity in citations in high-impact journal articles. JAMA Netw Open. (2021) 4(7):e2114509. 10.1001/jamanetworkopen.2021.14509 34213560 PMC8254129

[23] Merriman R Galizia I Tanaka S Sheffel A Buse K Hawkes S. The gender and geography of publishing: a review of sex/gender reporting and author representation in leading general medical and global health journals. BMJ Glob Health. (2021) 6(5). 10.1136/bmjgh-2021-005672 PMC8118011 33986001

[24] Shah SGS Dam R Milano MJ Edmunds LD Henderson LR Hartley CR Gender parity in scientific authorship in a National Institute for Health Research Biomedical Research Centre: a bibliometric analysis. BMJ Open. (2021) 11(3):e037935. 10.1136/bmjopen-2020-037935 PMC7993305 33757940

[25] Duma N Durani U Woods CB Kankeu Fonkoua LA Cook JM Wee C Evaluating unconscious bias: speaker introductions at an international oncology conference. J Clin Oncol. (2019) 37(36):3538–45. 10.1200/JCO.19.01608 31603705

[26] Williamson S. Backlash, gender fatigue and organisational change: AIRAANZ 2019 presidential address. Labour Ind. (2020) 30(1):5–15. 10.1080/10301763.2019.1677202

[27] Flood M Dragiewicz M Pease B. Resistance and backlash to gender equality. Aust J Soc Issues. (2021) 56(3):393–408. 10.1002/ajs4.137

[28] Wong BO Perera ND Shen JZ Turner BE Litt HK Mahipal A Analysis of registered clinical trials in surgical oncology, 2008–2020. JAMA Netw Open. (2022) 5(1):e2145511. 10.1001/jamanetworkopen.2021.45511 35084485 PMC8796015

[29] LaRocca CJ Wong P Eng OS Raoof M Warner SG Melstrom LG. Academic productivity in surgical oncology: where is the bar set for those training the next generation? J Surg Oncol. (2018) 118(3):397–402. 10.1002/jso.25143 30125359 PMC6160329

[30] Saunders CM Nichevich A Ellis C. Frontiers in academic surgery: the five M'S. ANZ J Surg. (2008) 78(5):350–5. 10.1111/j.1445-2197.2008.04473.x 18380729

[31] Kodadek LM Kapadia MR Changoor NR Dunn KB Are C Greenberg JA Educating the surgeon-scientist: a qualitative study evaluating challenges and barriers toward becoming an academically successful surgeon. Surgery. (2016) 160(6):1456–65. 10.1016/j.surg.2016.07.003 27524431

[32] Wells K Fleshman JW. Women in leadership. Clin Colon Rectal Surg. (2020) 33(4):238–42. 10.1055/s-0040-1712977 32624722 PMC7329381

[33] Heidari S Babor TF De Castro P Tort S Curno M. Sex and gender equity in research: rationale for the SAGER guidelines and recommended use. Res Integrity Peer Rev. (2016) 1(1):2. 10.1186/s41073-016-0007-6 PMC5793986 29451543

[34] van Diemen J Verdonk P Chieffo A Regar E Mauri F Kunadian V The importance of achieving sex- and gender-based equity in clinical trials: a call to action. Eur Heart J. (2021) 42(31):2990–4. 10.1093/eurheartj/ehab457 34352884 PMC8370758

[35] Kim ES Uldrick TS Schenkel C Bruinooge SS Harvey RD Magnuson A Continuing to broaden eligibility criteria to make clinical trials more representative and inclusive: ASCO-friends of cancer research joint research statement. Clin Cancer Res. (2021) 27(9):2394–9. 10.1158/1078-0432.CCR-20-3852 33563632 PMC12486295

[36] Shahid I Khan MS Sohail A Khan SU Greene SJ Fudim M Evaluation of representation of women as authors in pivotal trials supporting US food and drug administration approval of novel cardiovascular drugs. JAMA Netw Open. (2022) 5(2):e220035-e. 10.1001/jamanetworkopen.2022.0035 35212753 PMC8881772

[37] Van Spall HGC Lala A Deering TF Casadei B Zannad F Kaul P Ending gender inequality in cardiovascular clinical trial leadership: JACC review topic of the week. J Am Coll Cardiol. (2021) 77(23):2960–72. 10.1016/j.jacc.2021.04.038 34112322

[38] Cevik M Haque SA Manne-Goehler J Kuppalli K Sax PE Majumder MS Gender disparities in coronavirus disease 2019 clinical trial leadership. Clin Microbiol Infect. (2021) 27(7):1007–10. 10.1016/j.cmi.2020.12.025 33418021 PMC7785275

[39] Ludmir EB Mainwaring W Miller AB Lin TA Jethanandani A Espinoza AF Women’s representation among lead investigators of clinical trials in oncology. JAMA Oncol. (2019) 5(10):1501–2. 10.1001/jamaoncol.2019.2196 31393530 PMC6692670

[40] Muquith M Pham T Espinoza M Hsiehchen D. Representation of investigators by gender among authors of phase 3 oncology trials worldwide. JAMA Netw Open. (2022) 5(2):e220031. 10.1001/jamanetworkopen.2022.0031 35212754 PMC8881765

[41] Jou J Brodsky A Charo L Binder P Saenz C Eskander RN Trends and geographic variation in women's Representation as principal investigators (PI) in phase 3 gynecologic oncology clinical trials. Gynecol Oncol. (2021) 162(2):389–93. 10.1016/j.ygyno.2021.05.037 34099315

[42] Tregenza H. Snow Medical Research Foundation bars University of Melbourne from funding program after “six white men” sole recipients of honorary doctorates (2022). https://www.abc.net.au/news/2022-03-08/melbourne-uni-cut-off-from-snow-medical-funding/100891770?utm_campaign=abc_news_web&utm_content=link&utm_medium=content_shared&utm_source=abc_news_web

[43] Banerjee S Dafni U Allen T Arnold D Curigliano GMD Garralda E Gender-related challenges facing oncologists: the results of the ESMO Women for Oncology Committee survey. ESMO Open. (2018) 3(6):e000422. 10.1136/esmoopen-2018-000422 30273420 PMC6157518

[44] Shen MR Tzioumis E Andersen E Wouk K McCall R Li W Impact of mentoring on academic career success for women in medicine: a systematic review. Acad Med. (2022) 97(3):444–58. 10.1097/ACM.0000000000004563 34907962

[45] Farkas AH Bonifacino E Turner R Tilstra SA Corbelli JA. Mentorship of women in academic medicine: a systematic review. J Gen Intern Med. (2019) 34(7):1322–9. 10.1007/s11606-019-04955-2 31037545 PMC6614283

[46] Laver KE Prichard IJ Cations M Osenk I Govin K Coveney JD. A systematic review of interventions to support the careers of women in academic medicine and other disciplines. BMJ Open. (2018) 8(3):e020380. 10.1136/bmjopen-2017-020380 PMC5875640 29572397

[47] Morton B Vercueil A Masekela R Heinz E Reimer L Saleh S Consensus statement on measures to promote equitable authorship in the publication of research from international partnerships. Anaesthesia. (2022) 77(3):264–76. 10.1111/anae.15597 34647323 PMC9293237

[48] Bias TEsRiAG. The Editor's role in avoiding gender bias. Sci Ed. (2019) 42 85–96. https://www.csescienceeditor.org/article/the-editors-role-in-avoiding-gender-bias/#a-good-start

[49] Lundine J Bourgeault IL Glonti K Hutchinson E Balabanova D. “I don't see gender": conceptualizing a gendered system of academic publishing. Soc Sci Med. (2019) 235:112388. 10.1016/j.socscimed.2019.112388 31288167

[50] Luong V Segelov E. Gender representation in Australian academic Collaborative Cancer Clinical Trials Groups. Asia Pac J Clin Oncol. (2021) 17(59):60–109. 10.1111/ajco.1371532779388

